# Comparative genome analysis of the SPL gene family reveals novel evolutionary features in maize

**DOI:** 10.1590/1678-4685-GMB-2017-0144

**Published:** 2019-07-01

**Authors:** Xiaojian Peng, Qianqian Wang, Yang Zhao, Xiaoyu Li, Qing Ma

**Affiliations:** 1 National Engineering Laboratory of Crop Stress Resistance, Key Laboratory of Crop Biology of Anhui Province, School of Life Sciences, Anhui Agricultural University, Hefei, China; 2 Institute of Horticulture, Anhui Academy of Agricultural Sciences, Hefei, China

**Keywords:** SPL, phylogenetic relationship, gene duplication, miR156 expression

## Abstract

SPLs are plant-specific transcription factors that play important regulatory roles in plant growth and development. Systematic analysis of the SPL family has been performed in numerous plants, such as *Arabidopsis*, rice, and *Populus*. However, no comparative analysis has been performed across different species to examine evolutionary features. In this study, we present a comparative analysis of *SPLs* in different species. The results showed that 84 *SPLs* of the four species can be divided into six groups according to phylogeny. We found that most of the SPL-containing regions in maize showed extensive conservation with duplicated regions of rice and sorghum. A gene duplication analysis in maize indicated that *ZmSPLs* showed a significant excess of segmental duplication. The Ka/Ks analysis indicated that 9 out of 18 duplicated pairs in maize experienced positive selection, while SPL gene pairs of rice and sorghum mainly evolved under purifying selection, suggesting novel evolutionary features for *ZmSPLs*. The 31 *ZmSPLs* were further analyzed by describing their gene structure, phylogenetic relationships, chromosomal location, and expression, Among the *ZmSPLs,* 13 were predicated to be targeted by miR156s and involved in drought stress response. These results provide the foundation for future functional analyses of *ZmSPLs*.

## Introduction

Transcription factors (TFs) are a large class of regulators controling gene expression by activating or repressing target genes at the transcriptional level. Increasing evidence indicates that TFs have important roles in the regulating networks of plant growth and development processes (Riechmann *et al.*, 2001). SPLs (SQUAMOSA promoter binding protein-like) comprise a family of plant-specific transcription factors that contain a highly conserved SBP domain consisting of about 76 amino acids (Chen *et al.*, 2010). This domain has been implicated in DNA binding and nuclear localization, and also features two zinc-binding sites assembled as Cys-Cys-Cys-His and Cys-Cys-His-Cys, respectively (Klein *et al.*, 1996; Yamasaki *et al.*, 2004). Gene structure analysis indicated that the nuclear localization signal (NLS) region partially overlapped with the second Zn-finger located at the C-terminal of the SBP domain. SBP-domain encoding proteins were firstly isolated from *Antirrhinum majus* designated as AmSBP1 and AmSBP2. These two proteins can recognize a conserved motif in the promoter region of the floral meristem identity gene *SQUAMOSA*, which is a member of the MADS-box gene family based on its *in vitro* binding activity (Klein *et al.*, 1996). Subsequent experiments indicated that the palindromic GTAC core motif of the *cis-*element is essential for efficient DNA binding by different SBP proteins (Birkenbihl *et al.*, 2005; Cardon *et al.*, 1997). To date, the SPL gene family has been identified in various plant genomes, such as *Arabidopsis*, rice, and *Populus* (Cardon *et al.*, 1999; Xie *et al.*, 2006; Guo *et al.*, 2008; Li and Lu 2014).

In *Arabidopsis*, a total of 16 members have been identified as SPL proteins. Several biological experiments demonstrated that SPL proteins have important functions in plant development processes, especially flower development. For example, the *AtSPL3* gene was shown to be involved in the floral transition, and it was the first SPL gene identified in *Arabidopsis*. As an ortholog of *SQUAMOSA*, *AtSPL3* can interact with the promoter region of the floral meristem identity gene *APETALA1* (*AP1*), and constitutive expression of this gene in *Arabidopsis* can result in an early flowering phenotype (Cardon *et al.*, 1997). Loss-of-function mutation of the *Arabidopsis SPL8* gene indicated that *AtSPL8* can regulate pollen sac development (Unte *et al.*, 2003). In maize, the *tasselsheath4* (*tsh4*) mutant of an SPL gene was shown to regulate bract development and the establishment of meristem boundaries (Chuck *et al.*, 2010). In addition, SPL genes (*SPLs*) were also demonstrated to play crucial roles in fruit development (Manning *et al.*, 2006), leaf development (Stone *et al.*, 2005), plant hormone signaling (Zhang *et al.*, 2007), male fertility (Xing, 2010), and shoot development (Wu and Poethig, 2006).

Besides transcription factors, miRNAs are another other class of important regulators of gene expression, acting at the post-transcriptional level (Lee *et al.*, 1993; Zhang *et al.*, 2006). These small RNA molecules (20-24 nucleotides in length) can cause the degradation of mRNAs or repress translation by binding to the mRNAs of the target genes (Zhang *et al.*, 2006). Most of the miRNAs in plants are evolutionarily conserved, encoded by gene families (Jones-Rhoades *et al.*, 2006). Among them, miR156/157, a miRNA family that is highly conserved in plants (Axtell and Bowman, 2008), is thought to be involved in important developmental processes. Previous studies demonstrated that half of the SPLs have been found to be targeted by miR156/157 family. For example, 10 of the 16 *Arabidopsis* SPLs, were targeted by the miR156 family (Rhoades *et al.*, 2002; Schwab *et al.*, 2005; Wu and Poethig, 2006; Wang *et al.*, 2009; Yu *et al.*, 2010). In rice, 11 of the 19 SPLs were found to be regulated by OsmiR156 (Xie *et al.*, 2006).

Despite the progress in function studies of *SPLs* in many species, no comparative analysis has been reported across different species to study the evolution and functional relevance of this family. Although the maize SPL gene family has been reported by Hultquist and Dorweiler (Dorweiler, 2008), our understanding of this gene family in maize is still rather limited. Therefore, we firstly performed a comparative analysis of this family to dissect the evolutionary features in different species, and 31 *ZmSPLs* were further characterized, including gene structure, phylogenetic relationships, gene duplication, amongst others. Quantitative real-time PCR (RT-qPCR) analysis was performed to examine the expression pattern of miR156 targeted genes in different tissues and in response to drought stress. These results contribute to a basic understanding of the SPL gene family in different species, and provide a foundation to further elucidate the SPL gene function in maize.

## Material and Methods

### Whole-genome identification and phylogenetic analysis of *SPLs*

To identify maize SPL proteins, the Hidden Markov Model (HMM) profile of the SBP domain (PF03110.7) retrieved from Pfam database (http://pfam.xfam.org/) (Finn *et al.*, 2006) was adopted as query against maize genome database (http://www.maizesequence.org/index.html), with an cutoff E-value of le^-3^. Sequences of *Arabidopsis* and rice SPL proteins were also used to query against the maize genome to identify all possible maize SPL proteins (Cardon *et al.*, 1999; Xie *et al.*, 2006; Guo *et al.*, 2008). The candidate sequences that met the standards were confirmed again by Pfam database and SMART (http://smart.embl-heidelberg.de/) (Letunic, 2009). Finally, redundant sequences were removed manually after alignments using MUSCLE software (Edgar, 2004). To identify sorghum *SPLs*, the complete genome sequence of sorghum was obtained (ftp://ftp.ensemblgenomes.org/pub/plants/release-31/fasta/sorghum_bicolor/pep/), and the same method as described above was adopted. To understand the evolutionary relationships of the SPL family, full-length sequences of the SPL proteins were aligned using MUSCLE software. A phylogenetic tree was constructed using MEGA v4.0 (Tamura, 2007) by the neighbor-joining (NJ) method with 1,000 bootstrap replicates.

### Synteny analysis, gene duplication, and evolution analysis

Syntenic blocks among maize, rice, and sorghum were evaluated by MCScan software (Wang *et al.*, 2012) and alignments with an E-value of 1e^-5^ were considered significant matches. Then, the duplicated SPLs from these syntenic blocks were identified using a Perl script, and the relationships of the duplicated genes, including segmental and tandem duplications, were finally visualized using Circos (http://circos.ca) (Krzywinski *et al.*, 2009; Wang *et al.*, 2015). DnaSP v5.0 (Rozas *et al.*, 2003) was used to estimate the number of nonsynonymous substitutions per nonsynonymous site (Ka) and synonymous substitution per synonymous site (Ks) of the duplicated genes. The Ka/Ks ratios between duplicated genes were analyzed to deduce the selection model. To obtain further insight into selection pressure among duplicated gene pairs, a sliding window analysis of the Ka/Ks ratios was conducted with the following parameters: window size 150 bp and step size 9 bp. For duplication time analysis, the Ks value was translated into duplication time in million years based on a synonymous mutation rate of λ substitutions per synonymous site per year, as T = Ks/2λ10^-6^ million years ago (Mya) (λ= 6.510^-9^ for grasses) (Gaut *et al.*, 1996; Quraishi *et al.*, 2011).

### Sequence analysis and chromosomal locations of *ZmSPL* genes

Information regarding the exon number, open reading frame (ORF) length, molecular weight (kDa), and isoelectric point (pI) of maize SPL proteins were determined by the Expasy program (http://www.expasy.org/tools/). Gene structure was predicted through alignments of the coding sequences (CDS) with corresponding genomic sequences using GSDS (http://gsds.cbi.pku.edu.cn/) (Hu *et al.*, 2015). Conserved motifs were investigated by MEME (Multiple Expectation Maximization for Motif Elicitation) (Bailey and Elkan, 1995) with the parameters used in our previous study (Zhao *et al.*, 2011). The chromosome location image was generated by MapInspect software (http://www.plantbreeding.wur.nl/uk/software_mapinspect.html) according to the starting positions of *ZmSPLs* on the 10 chromosomes.

### Prediction of *ZmSPL* genes targeted by miR156

To predict *ZmSPLs* regulated by miR156, the sequence of maize miR156 was first obtained from miRBase (http://www.mirbase.org/) (Kozomara and Griffithsjones, 2010). Then, *ZmSPLs* targeted by miR156 were predicted by searching the coding regions and 3’ UTRs of all SPLs for complementary sequences to the maize miR156 sequence using psRNATarget server with default parameters (http://plantgrn.noble.org/psRNATarget/?function=3) (Dai and Zhao, 2011).

### Expression pattern analysis using transcriptome data

Transcriptome data of the genome-wide gene expression atlas of the maize inbred line B73 was used to elucidate the expression pattern of *ZmSPLs* during different development stages (Sekhon *et al.*, 2013). A heat map was generated based on the FPKM (fragments per kilobase of exon per million fragments mapped) values, which were initially transformed by taking log2 (FPKM+1) and then loaded into R and the Bioconductor program (http://www.bioconductor.org/) (Ross and Robert, 2008).

### Plant materials, stress treatments, RNA extraction, and RT-qPCR analysis

To examine the expression profile during different developmental stages, four representative tissues, including root, leaf, stem, and silk were collected from a life cycle of the maize inbred line B73. For stress treatment, maize seeds were surface-sterilized in 1 (v/v) Topsin-M (Rotam Crop Sciences Ltd.) for 10 min, washed in deionized water, and germinated on wet filter paper at 28 °C for 3 days. The germinated seeds were transplanted to enriched soil (turf to vermiculite in a ratio of 1:1) and grown in a greenhouse with a 14-h light/10-h dark cycle at 28-30 °C. Drought stress was performed by withholding watering at the three-leaf stage of maize seedlings. The seedling leaves were collected at 0, 1, 2, and 4 days after treatment with relative leaf water content (RLWC) decreased to 98, 70, 60, and 58%, which represented normal plants, slight, moderate, and severe stresses, respectively. For all the stages, three biological replicates were performed for each sample. For RNA isolation, all the collected samples were extracted using Trizol reagent (Invitrogen). To remove possible contaminating genomic DNA, the extracted RNAs were treated with DNase I (Invitrogen) for 20 min, then cDNAs were synthesized from 1 μg of total RNA using the PrimerScript RT Master mix (TaKaRa). For RT-qPCR analysis, gene-specific primers for maize SPL genes were designed using Primer Express 3.0 software (Applied Biosystems), listed in Table S1, and the PCR assays and data analysis were performed as described previously (Peng *et al.*, 2012).

Primer specificity were examined through the Primer Blast at NCBI, and their efficiency was tested by ordinary PCR. Amplification products were analyzed by agarose gel electrophoresis, and each primer pair was seen to amplified only one 100 bp product, which indicated that these primers were suitable for RT-qPCR. The RT-qPCRs were performed in an ABI 7300 Real-Time machine, with a total reaction volume of 20 μL, containing SYBR Green Master Mix reagent, cDNA sample, primers and RNase-free water. The PCR run program was as follows: denaturation (95 °C for 10 min), amplification and quantification (40 cycles of 95 °C for 15 s and 60 °C for 1 min), melting curve analysis (60–95 °C, with a heating rate of 0.3 °C/s). The *ZmActin* gene was used for data normalization, and for each sample three technical replicates were performed. Relative expression levels were calculated using the comparative delta delta cycle threshold (ΔΔct) method. The SPSS 19.0 software (http://www.spss.com.cn/) was used for statistical analysis.

## Results

### *SPL* genes in different species

In previous studies, a total of 19, 16, and 31 *SPLs* were identified in rice, *Arabidopsis*, and maize, repectively (Cardon *et al.*, 1999; Xie *et al.*, 2006; Dorweiler, 2008; Guo *et al.*, 2008). Due to maize genome database updates, we performed a BlastP search against the genome database to identify maize *SPLs* using the Hidden Markov Model (HMM) profile of the SPL domain, and the same strategy was used to identify sorghum *SPLs*. By this approach, a total of 31 and 18 non-redundant sequences in maize and sorghum were identified after searching against Pfam and SMART, respectively. The total number of *SPLs* in maize was the same as in a previous study. In addition, the number of sorghum *SPLs* was similar to that in rice and *Arabidopsis*. These genes were named *ZmSPL1*–*ZmSPL31* and *SbSPL1*–*SbSPL18* according to their order of distribution on the chromosomes (Tables S2, S3). It should be noted that the number of *SPLs* in the maize genome was greater than that in rice, *Arabidopsis*, and sorghum. This gives rise to the question, as to where did these additional genes originally come from in the maize genome. To elucidate the possible mechanism(s) of this phenomenon, we subsequently performed a comparative analysis of SPL gene family in these species.

### Phylogenetic relationships of SPLs

**Figure 1:**
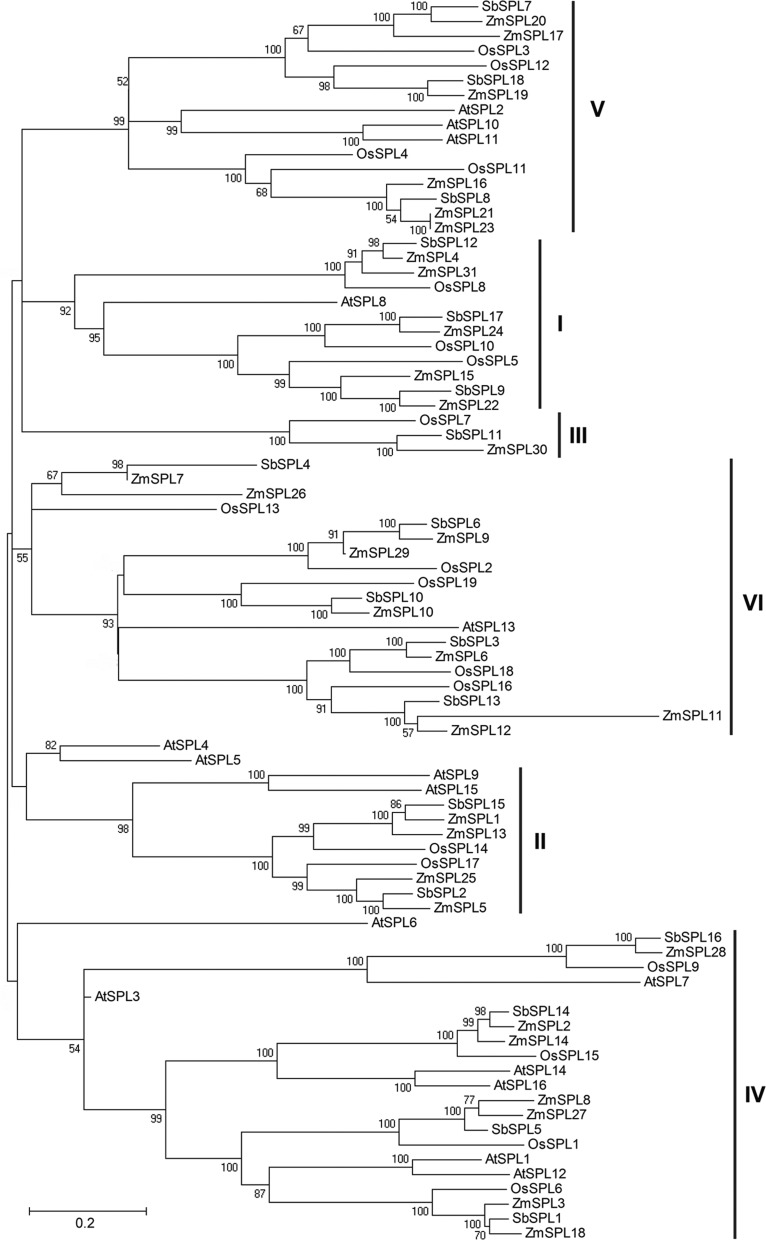
Phylogenetic relationships of maize, rice, sorghum, and *Arabidopsis* SPL proteins. The phylogenetic tree was constructed using MEGA4.0 with the NJ method. Bootstrap values above 50% are shown at each node.

To examine the evolutionary relationships of *SPLs* among different plant species, full-length sequences of the SPL proteins were aligned using MUSCLE, and then a combined phylogenetic tree of 84 SPL protein sequences from the four species, including 31 of maize, 19 of rice, 18 of sorghum, and 16 of *Arabidopsis*, was constructed using the NJ method with 1000 bootstrap replicates (Figure 1). The 84 *SPLs* were divided into six subfamilies (I-VI) according to phylogenetic relationship (bootstrap value > 50%). Although each of the subfamilies contained representative of rice, sorghum, and *Arabidopsis SPLs*, most maize *SPLs* showed closer relationships with sorghum *SPLs* than rice and *Arabidopsis*, suggesting a closer evolutionary relationship of the two species. For example, a total of 16 orthologous pairs were identified between maize and sorghum. We noted that the number of *SPLs* located in different subfamilies had a significant difference, ranging from 3 (III) to 20 (IV). Most of the members located in the same phylogenetic clade had well-supported bootstrap values, while some proteins showed unclear evolutionary relationships with lower bootstrap values, such as *AtSPL4*, *AtSPL5*, and *AtSPL6*. We also noted that the numbers of maize SPL proteins in most of the six groups were higher than other species, suggesting *SPLs* had especially expanded in the maize genome.

### Synteny analysis of *SPLs* among maize, sorghum, and rice

**Figure 2:**
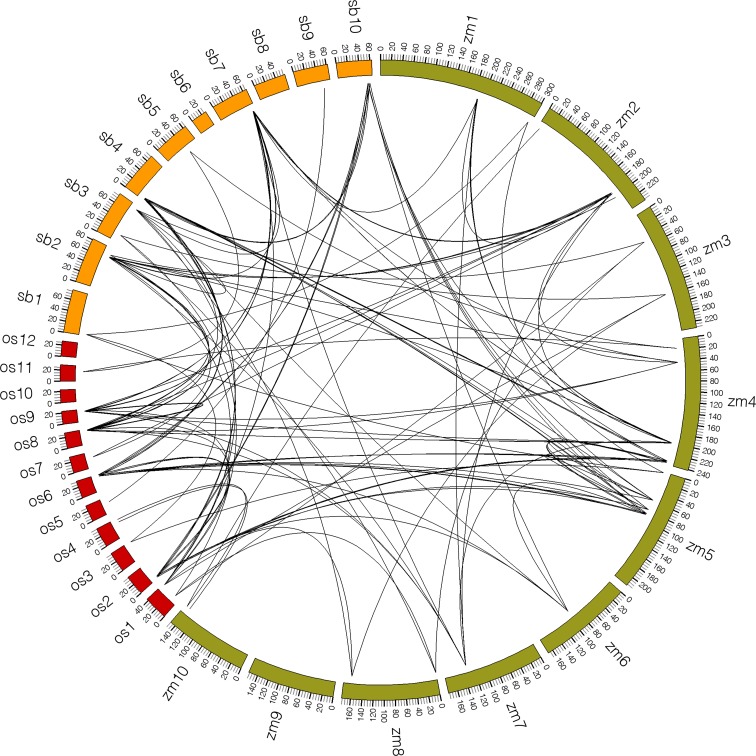
Synteny analysis of 68 *SPLs* from maize, sorghum, and rice. Maize, sorghum and rice chromosomes were labeled zm, sb, and os by different color boxes, respectively. The numbers along each chromosome box indicate sequence length of each chromosome in megabases. Black lines represent the syntenic relationships of orthologous gene pairs.

To examine the origin and evolutionary history of *SPLs* among maize, sorghum, and rice, a comparative analysis was performed to identify SPL orthologous pairs. Because *Arabidopsis* belongs to the Dicotyledoneae group of plants, orthologous pairs were not detected with the three other species. Through the comparative analysis of the genomic regions hosting the *SPLs* using MCScan software, we observed strongly conserved synteny among the three species. A total of 104 orthologous gene pairs were found among maize, rice, and sorghum, including 38 pairs between maize and rice, 36 pairs between maize and sorghum, and 30 pairs between sorghum and rice (Figure 2, Table S4). The numbers of orthologous gene pairs among the three plants were similar, suggesting the conserved evolution of the SPL family. Some differences were also observed among the three species, for example, the *ZmSPL16* and *ZmSPL17* had two orthologous genes in rice (*ZmSPL16/OsSPL4, OsSPL11*; *ZmSPL17/OsSPL3*, *OsSPL12*), while only one was identified in sorghum (*ZmSPL16/SbSPL8*; *ZmSPL17/SbSPL7*), respectively, which might be related to gene loss in the evolution of sorghum. In addition, the syntenic information also provided important clues to study the putative function of the collinear gene. For example, *ZmSPL4* encoding the *lg1* gene (Moreno *et al.*, 1997) had one collinear gene in rice (*OsSPL8*) as well as in sorghum (*SbSPL12*). Especially, *ZmSPL11* encoding the *tga1* gene (Wang *et al.*, 2005) had two orthologous genes in rice (*OsSPL16* and *OsSPL18*) and sorghum (*SbSPL3* and *SbSPL13*). These genes existing in different species might have originated from a common ancestor, which might share a similar regulatory role in plant growth and development.

### Gene duplication of *SPLs*

**Figure 3:**
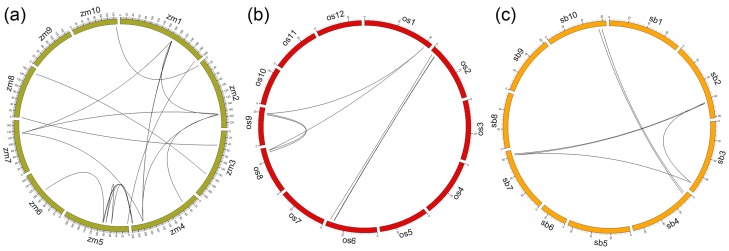
Synteny analysis of maize (a), rice 9 (b), and sorghum (c) SPLs. Maize, sorghum, and rice chromosomes were labeled zm, sb and os by different color boxes, respectively. The number along each chromosome box indicate sequence length of each chromosome in megabases. Black lines represent the syntenic relationships between SPLs.

The number of *ZmSPLs* (31) was almost twice that of *Arabidopsis* (16), and also much higher than that in rice (19) and sorghum (18) (Cardon *et al.*, 1999; Xie *et al.*, 2006). Gene duplication, including tandem and segmental duplications, are thought to have played important roles in the amplification of gene families in animals and plants (Moore and Purugganan, 2003). Thus, potential duplication events were analyzed to reveal the mechanism(s) behind the expansion of the maize SPL family. According to the syntenic regions and phylogenetic analysis, 18 *ZmSPL* gene pairs (24 genes) were located on the segmental duplication regions, accounting for 77.4% of the number of *ZmSPLs* (Figure 3, Table 1). In sorghum, six gene pairs (nine genes) were localized on the segmental duplication regions, accounting for 50% of the sorghum *SPLs*. In rice, 11 members forming seven gene pairs were detected, which accounted for 57.8% of the rice *SPLs*. In addition, no significant tandem duplication events were detected among the three plants. These findings indicated that segmental duplication was the major factor that contributed to the expansion of SPL gene family, especially for maize.

**Table 1:** Ka/Ks analysis and estimated divergence time for the duplicated SPL paralogs

Duplicated pairs	Ka	Ks	Ka/Ks	Purifying selection	Date (Mya)	Duplicate type
*ZmSPL1-ZmSPL13*	0.135	0.164	0.822	Yes	12.61	Segmental
*ZmSPL5-ZmSPL25*	0.122	0.126	0.966	Yes	9.68	Segmental
*ZmSPL1-ZmSPL5*	0.394	0.395	0.997	Yes	30.39	Segmental
*ZmSPL1-ZmSPL25*	0.374	0.344	1.088	No	26.45	Segmental
*ZmSPL13-ZmSPL5*	0.346	0.651	0.532	Yes	50.08	Segmental
*ZmSPL13-ZmSPL25*	0.373	0.279	1.337	No	21.45	Segmental
*ZmSPL2-ZmSPL14*	0.100	0.063	1.605	No	4.81	Segmental
*ZmSPL3-ZmSPL18*	0.083	0.073	1.136	No	5.64	Segmental
*ZmSPL4-ZmSPL31*	0.145	0.093	1.559	No	7.14	Segmental
*ZmSPL6-ZmSPL11*	0.480	0.565	0.849	Yes	43.49	Segmental
*ZmSPL8-ZmSPL27*	0.114	0.122	0.934	Yes	9.35	Segmental
*ZmSPL9-ZmSPL29*	0.124	0.100	1.242	No	7.65	Segmental
*ZmSPL15-ZmSPL22*	0.173	0.385	0.449	Yes	29.63	Segmental
*ZmSPL22-ZmSPL24*	0.365	0.611	0.598	Yes	46.98	Segmental
*ZmSPL16-ZmSPL21*	0.101	0.085	1.178	No	6.56	Segmental
*ZmSPL17-ZmSPL20*	0.257	0.272	0.948	Yes	20.88	Segmental
*ZmSPL17-ZmSPL19*	0.553	0.526	1.051	No	40.46	Segmental
*ZmSPL20-ZmSPL19*	0.440	0.394	1.118	No	30.30	Segmental
*SbSPL2-SbSPL15*	0.228	0.467	0.488	Yes	35.923	Segmental
*SbSPL3-SbSPL6*	0.406	0.534	0.760	Yes	41.08	Segmental
*SbSPL3-SbSPL13*	0.206 0.2061	0.474	0.435	Yes	34.46	Segmental
*SbSPL6-SbSPL13*	0.426	0.597	0.714	Yes	45.92	Segmental
*SbSPL18-SbSPL7*	0.265	0.611	0.433	Yes	47.00	Segmental
*SbSPL17-SbSPL9*	0.306	0.419	0.730	Yes	32.23	Segmental
*OsSPL2-OsSPL16*	0.380	0.519	0.732	Yes	39.92	Segmental
*OsSPL2-OsSPL18*	0.496 0.496	0.496	1.000	No	38.15	Segmental
*OsSPL3-OsSPL12*	0.487	0.524	0.929	Yes	40.31	Segmental
*OsSPL4-OsSPL11*	0.317	0.535	0.593	Yes	41.15	Segmental
*OsSPL5-OsSPL10*	0.338	0.412	0.820	Yes	31.69	Segmental
*OsSPL14-OsSPL17*	0.185	0.450	0.411	Yes	34.62	Segmental
*OsSPL16-OsSPL18*	0.292	0.461	0.633	Yes	35.46	Segmental

**Figure 4:**
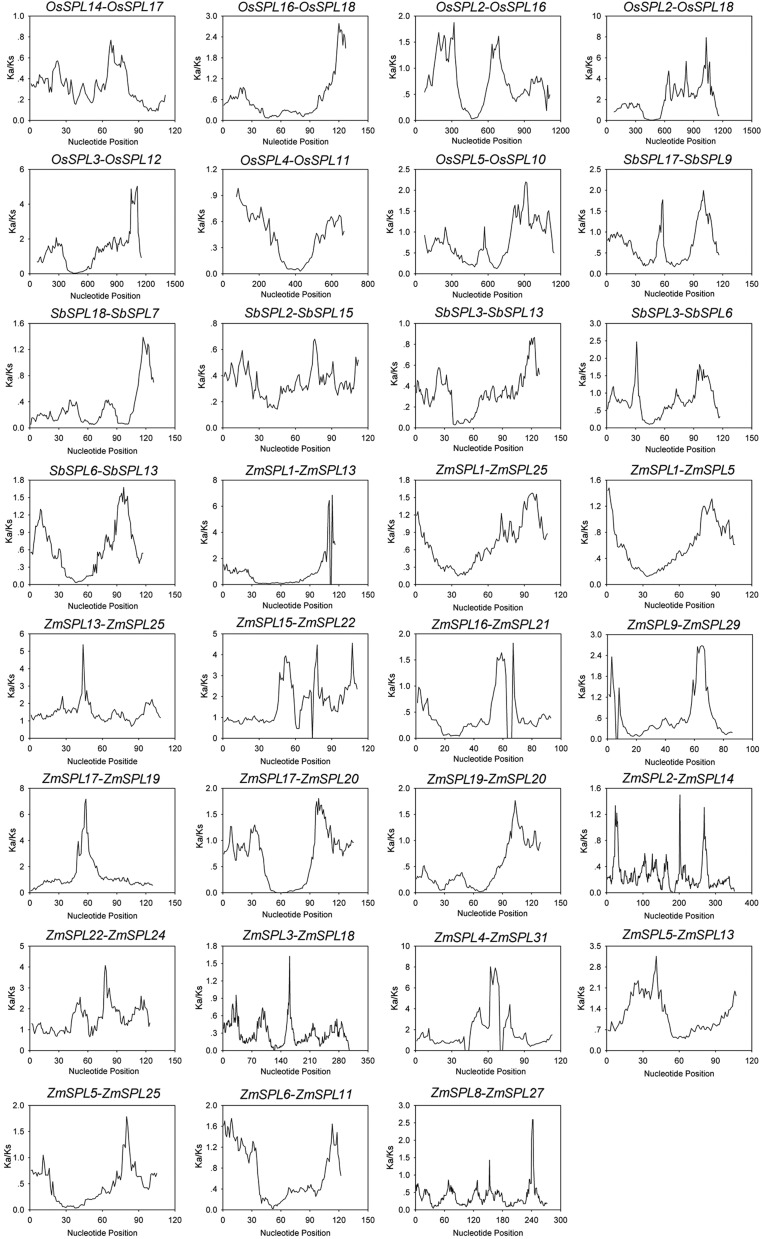
Sliding window plots of segmental duplicated SPLs. Window size is 150 bp, and step size is 9 bp.

To further understand the duplication and divergence of *SPLs*, the Ka, Ks, and Ka/Ks ratio were calculated for each duplicated pair. The Ka and Ks results were used to examine the course of divergence after duplication, and the Ka/Ks ratio was applied to explore different selective constrains. Generally, a Ka/Ks ratio < 1 means purifying selection, a ratio = 1 indicates neutral selection, while a ratio > 1 stands for positive selection (Lynch and Conery, 2000). The results showed that the Ka/Ks ratio of the 18 duplicated *ZmSPLs* pairs ranged from 0.449 to 1.605. Among them, nine duplicated pairs had a Ka/Ks ratio <1. Moreover, the values of *ZmSPL13/-5*, *ZmSPL15/-22* and *ZmSPL22/-24* were less than 0.6, which suggests strong purifying selection during evolution. The other nine pairs showed a Ka/Ks ratio >1, indicating that these gene pairs evolved under positive selection (Table 1). In rice and sorghum, the Ka/Ks ratios of all gene pairs were < 1, except for *OsSPL2*/*-18*, suggesting that these gene pairs mainly evolved under purifying selection. To obtain further insight into the selection pressure of different sites/regions, we performed a sliding-window analysis of the Ka/Ks ratio for each duplicated gene pair. As shown in Figure 4, numerous sites/regions showed evidence of strong positive selection, especially for *ZmSPL* gene pairs. In contrast, the other sites/regions were conserved under purifying selection, such as *OsSPL14*/-*17* and *SbSPL2*/-*15*.

According to the estimation for Ks, the dates for 31 segmental duplication pairs of maize, rice, and sorghum, were calculated based on a rate of 6.5 10^-9^ substitutions per site per year (Gaut *et al.*, 1996; Quraishi *et al.*, 2011). The results indicated that the 18 maize duplication events were estimated to have occurred approximately between 4.81 to 50.08 Mya (Table 1), and the duplication events of rice and sorghum *SPLs* were estimated to have occurred between 34.46 to 47.00 Mya.

### Sequence analysis of maize *SPLs*

Molecular weight (MW) and isoelectric point (pI) of the 31 *ZmSPLs* were determined using the Expasy server. The results showed that the ZmSPL proteins had a large variation in the length (bp) of the open reading frame (ranging from 300 to 3,339 bp) (Table S2). The 31 *ZmSPLs* were divided into six subfamilies based on the unrooted NJ tree (Figure S1a). Gene structure analysis indicated that the maize SPL family had highly diverse distributions of exon regions (Figure S1b). However, most *SPLs* within the same subfamilies of the phylogenetic tree had a similar gene structure. A total of 20 conserved motifs were identified in the maize SPL proteins (Table S5). Compared with the phylogenetic analysis, we found that genes located in the same subfamily had similar motif compositions (Figure S2). According to the starting positions of the maize SPL genes annotated by the maize B73 genome database, chromosome location analysis indicated that all of the 31 *ZmSPLs* were mapped to 9 of the 10 chromosomes with a clear non-random distribution (Figure S3) with approximately 45% of the *SPLs* on chromosome 4 (eight genes) and 5 (six genes).

### Identification of *ZmSPLs* targeted by miR156

**Figure 5:**
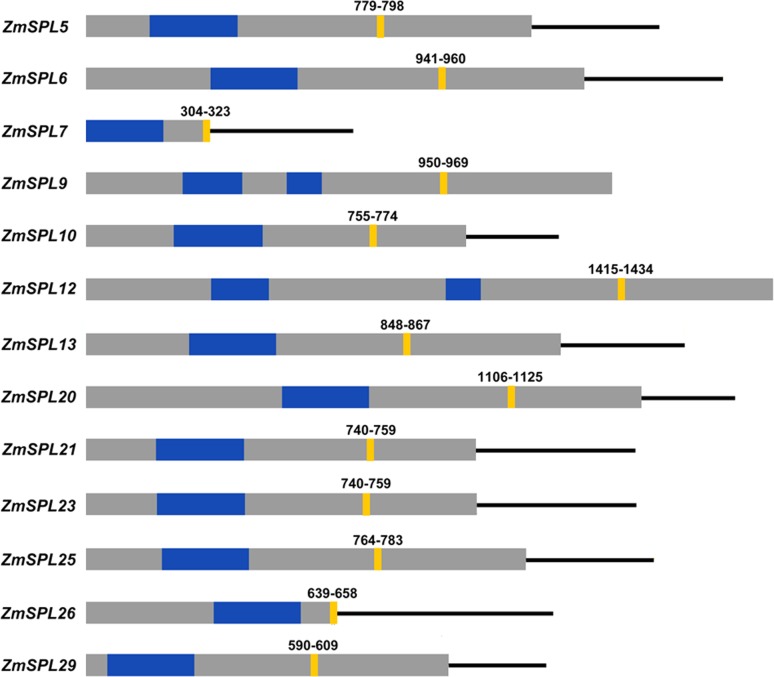
*ZmSPLs* targeted by miR156. Open reading frames (ORFs) are indicated by grey rectangles, the SBP domain is shown by blue rectangles, and the lines flanking ORFs represent 3’ UTRs. miR156 targeting sites are indicated by yellow rectangles.

**Figure 6:**
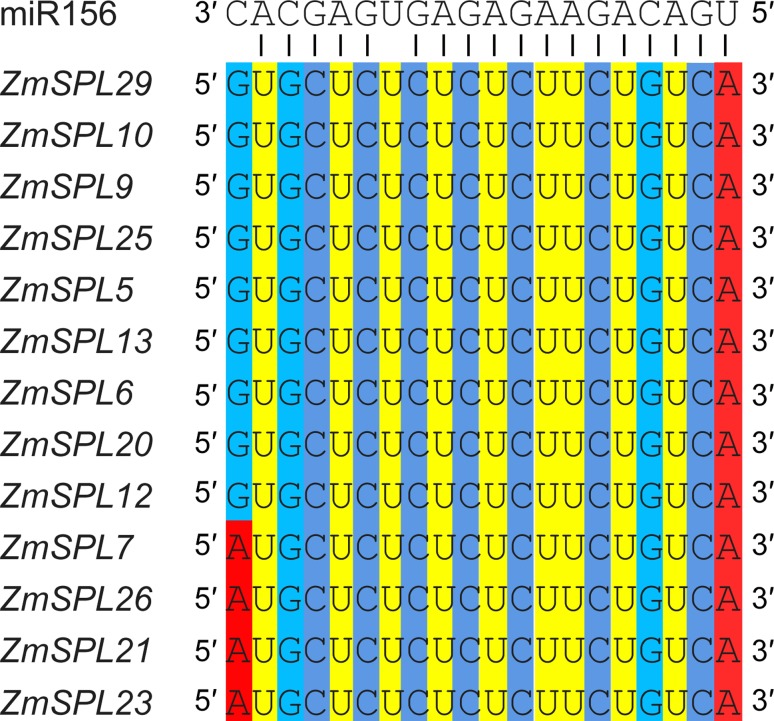
Sequence alignments of maize miR156 with their complementary sequence in the coding sequences and 3’ UTRs of *ZmSPLs*.

A series of *SPLs* have been confirmed to be targeted by miR156 in *Arabidopsis*, grape, and *Populus*. In general, the complementary sites of miR156 tend to be completely conserved and to locate in the coding regions or 3’ UTRs of *SPLs* in different plants (Schwarz *et al.*, 2008; Hou *et al.*, 2013; Li and Lu, 2014;). To identify the *ZmSPLs* targeted by miR156, we searched the coding regions and 3’ UTRs of all *ZmSPLs* for targets of maize miR156 using the psRNATarget online prediction tool with default parameters (Dai and Zhao, 2011). A total of 13 *ZmSPLs* were predicted to be potential targets of miR156 (Figure 5). We also found that the targeting sites of miR156 were located in coding regions for 11 *ZmSPLs*, and only two complementary sites were located in the 3’ UTRs (*ZmSPL7* and *ZmSPL26*). Consistent with previous studies, the targeting sites of maize SPLs were highly conserved in the evolution by the alignments of miR156 with their complementary sequence of maize SPLs (Figure 6).

### Expression patterns of *ZmSPL* genes in different developmental stages

**Figure 7:**
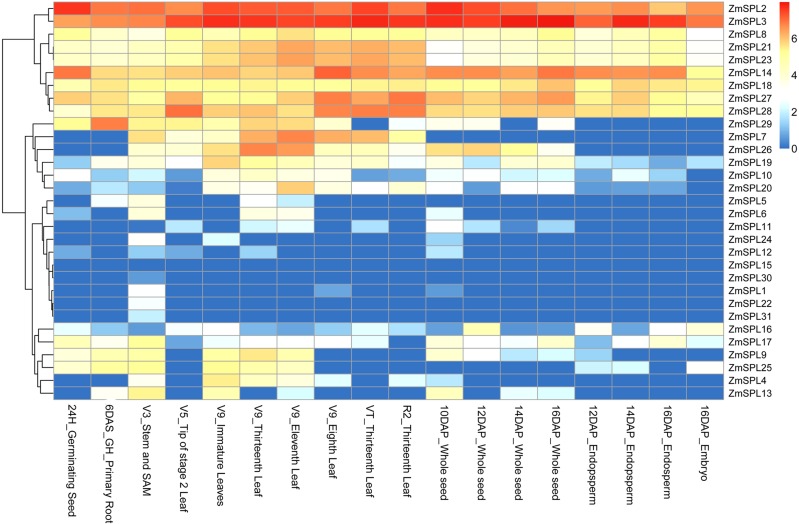
Expression profiles of *ZmSPLs* at different developmental stages. Blue and red indicate low and high levels of transcript abundance, respectively. Tissues from different developmental stages are shown at the bottom of the heat map.

The transcriptome data of the genome-wide gene expression atlas of maize was used to analyze the expression patterns of *SPLs* in different developmental stages (Sekhon *et al.*, 2013) (Figure 7). The results showed that most *ZmSPLs* had ubiquitously expression in the 18 different tissues. The group IV members seem to play regulatory roles in maize at multiple development stages based on the constitutive expression at relatively high level in all of the 18 tissues. On the contrary, the group I genes were only expressed in one or a few tissues and at a very low expression level, for example, *ZmSPL22* and *ZmSPL31* are merely expressed in V3_Stem and SAM. Furthermore, *ZmSPL15* was not expressed among the 18 tissues. By comparing the expression patterns of the duplicated gene pairs, we found that most of the duplicated gene pairs had similar expression patterns, but some with obvious divergence were also observed. For example, *ZmSPL31* is only expressed in V3-Stem and SAM, while its paralog *ZmSPL4* is expressed in V3-Stem and SAM, different stages of leaf and 10-DAP whole seed.

**Figure 8:**
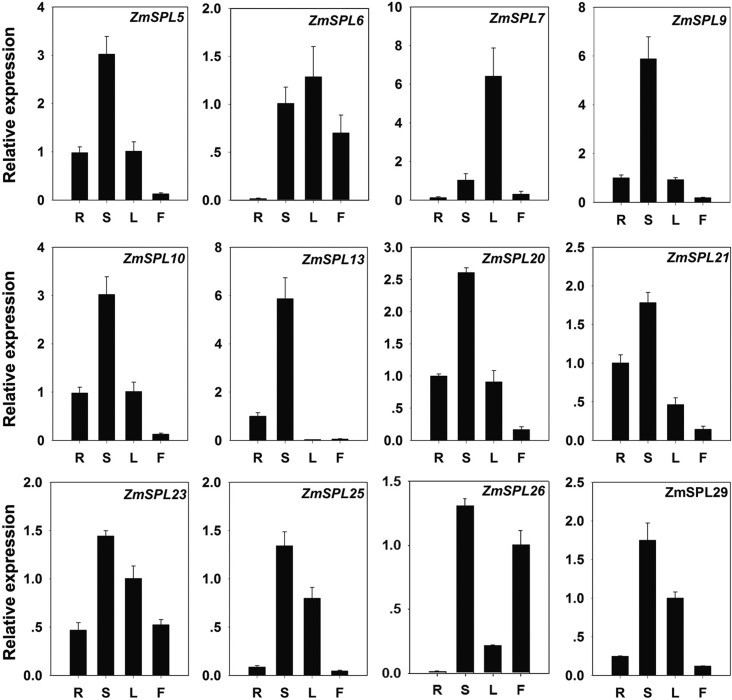
Expression patterns of 12 miR156 targeted *ZmSPLs* in four representative tissues. R: root; S: stem; L: leaf; F: filament. Shown are means ± SE.

The expression patterns of the 13 *ZmSPLs* targeted by miR156 were further investigated by quantitative real-time PCR (RT-qPCR) in different tissues. Four representative tissues, including root, leaf, stem, and silk were used in this study. A total of 12 genes were detected in the four tissues (*ZmSPL12* was not detected), and different expression levels were found. Most of the genes showed high expression in stem or leaves, especially *ZmSPL5*, *ZmSPL7*, *ZmSPL9*, *ZmSPL10*, and *ZmSPL13*. We also noted that segment duplicated genes had similar expression patterns of, for example *ZmSPL5* and *ZmSPL13*, suggesting conserved evolution in maize (Figure 8).

### Expression patterns of *ZmSPL* genes under drought stress

**Figure 9:**
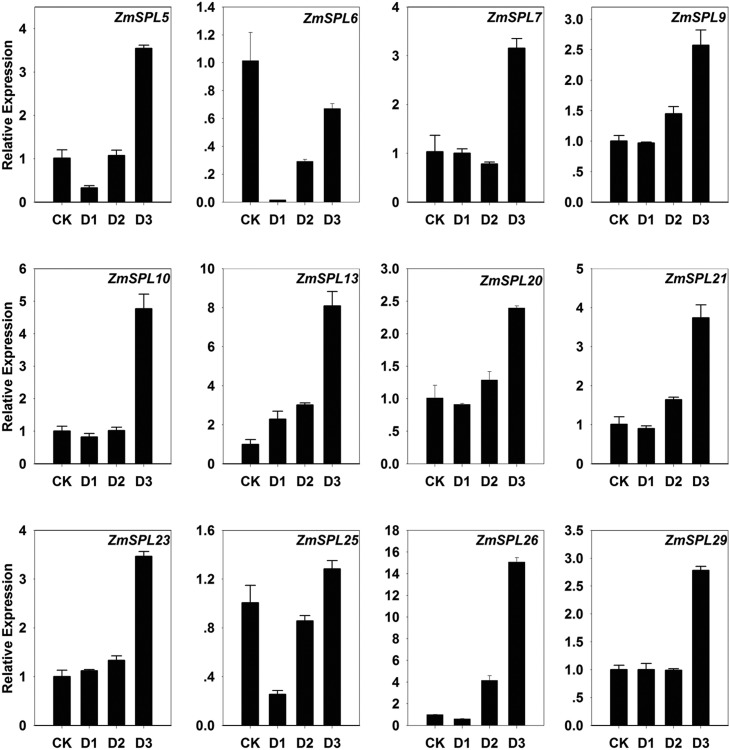
Expression patterns of 12 miR156 targeted *ZmSPLs* under drought stress. CK: normal plant; D1: slight stress; D2: moderate stress; D3: severe stress. Shown are means ± SE.

While most studies so far focused on divergent biological processes regulated by SPL genes, increasing evidence indicates that *SPLs* have also important roles in the response to abiotic stresses (Hou *et al.*, 2013; Wang *et al.*, 2009). To identify the possible members of *ZmSPLs* involved in drought stress, the expressions of the 13 miR156 targeted genes were further examined by RT-qPCR in maize leaves under slight, moderate, and severe stress (Figure 9). Consistent with the results of the expression at different developmental stages, the expression of *ZmSPL12* was not detected, and all of the other 12 genes were responsive to drought stress, suggesting important functions in stress regulation. Among the 12 genes, the highest expression level was observed under severe stress treatment, especially for *ZmSPL10*, *-13*, *-21*, and *-26*. In addition, the segment with duplicated genes showed similar expression patterns, which might suggest their redundant function in the regulation of maize drought response.

## Discussion

*SPLs* encode a large gene family of plant-specific transcription factors that play crucial roles in plant growth and development (Klein *et al.*, 1996; Cardon *et al.*, 1997). In the present study, we performed a comparative analysis of the SPL family to examine the evolutionary history in different species, thus providing a foundation for gene function analysis. At least 16 *SPLs* were reported in *Arabidopsis*, 19 in rice, and 28 in *Populus* (Cardon *et al.*, 1999; Xie *et al.*, 2006; Li and Lu, 2014). In this study, a total of 31 and 18 *SPLs* were identified in maize and sorghum, respectively. The phylogenetic tree of the 84 SPL proteins, including 31 of maize, 19 of rice, 18 of sorghum, and 16 of *Arabidopsis*, were divided into six groups. It should be noted that the number of maize *SPLs* was much higher than that in the mentioned species. With the purpose of elucidating the expansion mechanism of the maize SPL family, gene duplication events were investigated, which are thought to have occurred during the process of evolution. Generally, gene duplications were major driving forces in the evolution of genomes, and played vital roles in the expansion of gene families in various species (Moore and Purugganan, 2003; Mehan, 2004; Cannon *et al.*, 2004), such as NBS, HD-Zip, PHD, and others (Zhao *et al.*, 2011; Cheng *et al.*, 2012; Wang *et al.*, 2015).

According to the phylogenetic relationships and synteny analysis, a total of 18 segmental duplicate gene pairs of maize *SPLs* were identified, which accounted for 77.4% of maize SPL family genes. However, only 50% and 57.8% of the sorghum and rice *SPLs*, respectively, were detected to be involved in segmental duplication. Among the 68 *SPLs* of the three species, no tandem duplication events were detected. Thus, the segmental duplication was largely responsible for the expansion of SPL gene family. By comparing the frequency of segmental duplication in the three species, the segmental duplication of maize *SPLs* was seen to be more prevalent than in the sorghum and rice genomes, which provided a possible reason or explanation for why the numbers of *SPLs* are significantly different among maize, rice, and sorghum. In general, tandem duplication often occurred in rapidly evolving gene families, while segmental duplication was commonly reported in more slowly evolving gene families, *e.g.* the HD-Zip gene family (Cannon *et al.*, 2004; Guo *et al.*, 2008; Zhao *et al.*, 2011). We concluded that the prevalence of segmental duplication demonstrated the slow evolutionary rate of the SPL gene family. In fact, a total of 38 orthologous gene pairs were identified between maize and rice, which was similar with the result between maize and sorghum (36), as well as between rice and sorghum (30). Therefore, these results suggested that the SPL gene family is a highly conserved and slowly evolving family in plants.

Whole-genome duplication (WGD) played crucial roles in plant diversification and evolution, and was often accompanied by polyploidization and gene loss (Otto and Whitton, 2000; Soltis *et al.*, 2009). Previous studies showed that grass species have undergone several rounds of WGD. For example, maize experienced an ancient duplication prior to the divergence of grasses at approximately 50-70 Mya and a additional WGD at approximately 5 Mya, which separated maize from sorghum (Gaut, 2002; Salse *et al.*, 2008; Schnable *et al.*, 2009). The duplication time for the 18 ZmSPL segmental duplication pairs ranged from 4.81 to 50.08 Mya. Among them, seven pairs showed a duplication time of less than 10 Mya. However, all the segmental duplication events in the rice and sorghum genomes were shown to have occurred between 34.46 to 47.00 Mya. These results suggested that some segmental gene pairs of maize *SPLs* are due to a recent duplication. In addition, selection pressure analysis indicated that 50% of the maize duplicated pairs evolved under positive selection. Unlike in maize, SPL gene pairs of rice and sorghum mainly evolved under purifying selection, indicating novel evolutionary features of maize *SPLs*.

miR156 is one of the miRNA families that is highly conserved and functions in diverse processes associated with growth and development. It has been shown to mediate posttranscriptional regulation for a subset of SPLs through direct cleavage (Wu *et al.*, 2009; Yu *et al.*, 2010). For example, previous studies have identified 10, 11, and 18 potential SPLs as the targets of miR156 in rice, *Populus*, and tomato, respectively (Wu and Poethig, 2006; Xie *et al.*, 2006; Addoquaye *et al.*, 2008; Schwarz *et al.*, 2008; Li and Lu, 2014). In this study, 13 of 31 *ZmSPLs* contained miR156 recognition sites. It is noteworthy that *ZmSPL1* and *ZmSPL17* are not regulated by miR156, while their duplicated genes *ZmSPL13* and *ZmSPL20* are targets of miR156. This finding suggested that some distinct regulatory mechanisms might exist in these duplicated genes. In most cases, the miR156-regulated *SPLs* are master regulators that play divergent and redundant roles in plant morphology and development (Schwab, 2012). For example, *AtSPL3*, *AtSPL4*, and *AtSPL5* are mainly involved in the regulation of floral development (Cardon *et al.*, 1997; Jung *et al.*, 2011), while *AtSPL2*, *AtSPL10*, and *AtSPL11* have been shown to be involved in lateral organ development in the reproductive phase (Shikata *et al.*, 2009). However, whether the miR156-regulated *ZmSPLs* have similar regulatory roles remains to be further confirmed experimentally.

According to the microarray expression profile analysis, we found that some duplicated gene pairs have similar expression patterns, suggesting that the duplicated genes might have redundant functions in plant growth and development. Exceptions to this were also observed. The phylogenetic analysis showed that most of the maize SPL duplicated gene pairs located in the same branch had a high bootstrap value, and the duplicated gene pairs also exhibited similar exon/intron distribution and motif components. However, some duplicated gene pairs were shown to have significant divergence in expression patterns, such as *ZmSPL31* and *ZmSPL4*. These results suggested that most of the duplicated gene pairs were still conserved in their evolution, but that functional diversification has also accompanied the evolutionary process, as a major feature of retained duplicated genes in long-term evolution (Blanc and Wolfe, 2004). The expression patterns of the 12 miR156-targeted genes were further investigated at different developmental stages by RT-qPCR. Among the 12 *ZmSPLs*, high expression was detected in leaf and stem. Especially, the results confirmed that some segment duplicated genes have similar expression patterns, suggesting their conserved evolution and redundant functions. The expression of the 12 *ZmSPLs* under drought stress was also examined. Since most of the studies about SPL family were related to developmental and biological processes, this result provided important information that the 12 miR156 targeted genes are involved in drought stress, which may have important implications in revealing the function and mechanism of *SPL* in the stress response.

With the advances of sequencing technologies, many new miRNAs have been identified, and an increasing number of studies on miRNAs are being reported. miR156-based regulation of SPL genes participates in various biological pathways and has been reported in many plants, such as *Arabidopsis*, rice and others, but nearly no research is reported in maize. Based on our experimental results, we have identified several drought-response genes and cloned them, and this will be further studied by transgenic technology. In addition, we are verifying the actual regulatory relationship between miRNA156 and these cloned genes by 5’ RACE technology and degradation group sequencing technology, and we hope our research will reveal a new molecular mechanism in the maize abiotic stress response.

## Supplementary material

The following online material is available for this article:

Figure S1 Phylogenetic relationships and gene structure of the *ZmSPL*s.

Figure S2 Distribution of conserved motifs identified in the putative SPL proteins.

Figure S3 Chromosomal locations of *ZmSPLs* on the 10 maize chromosomes.

Table S1 List of gene-specific primers used in the present study.

Table S2 Detailed information on the 31 *SPLs* in the maize genome.

Table S3 Detailed information on the 18 sorghum *SPLs*.

Table S4 Information about orthologous genes in maize, rice, and sorghum.

Table S5 Detailed information on the 20 motifs identified in *ZmSPLs*.
