# Supplementary material for: Comparative genome analysis of the SPL gene family reveals novel evolutionary features in maize
Source: Genet Mol Biol. 2019 Jul 1;42(2):380–94. doi: 10.1590/1678-4685-GMB-2017-0144 (PMC6726161; doi:10.1590/1678-4685-GMB-2017-0144)
Supplement: Supplementary file 2 [file 1415-4757-GMB-1678-4685-GMB-2017-0144-suppl5.pdf]

Supplementary Material to "Comparative genome analysis of the SPL gene family reveals novel evolutionary features in maize"

Table S2 - Detailed information of the 31 *SPLs* in the maize genome

Gene Name[a]	Ensembl transcript ID	ORF (bp)[b]	Introns	Deduced polypeptide[c]		
				Length(aa)	MW(kDa)	pI
ZmSPL1	GRMZM2G160917_P02	1212	2	403	41.67	8.73
ZmSPL2	GRMZM2G169270_P01	3321	9	1106	121.17	6.97
ZmSPL3	GRMZM2G156756_P01	2919	10	972	105.91	5.43
ZmSPL4	GRMZM2G036297_P01	1200	2	399	43.36	7.45
ZmSPL5	GRMZM2G126018_P01	1152	2	383	40.20	8.64
ZmSPL6	GRMZM2G061734_P01	1290	2	429	44.96	6.73
ZmSPL7	GRMZM2G113779_P01	300	1	99	11.29	10.05
ZmSPL8	GRMZM2G133646_P01	2646	10	881	98.61	7.27
ZmSPL9	GRMZM5G878561_P01	1344	3	447	46.72	9.21
ZmSPL10	GRMZM2G106798_P01	978	2	325	34.38	8.70
ZmSPL11	GRMZM2G101511_P02	1395	2	464	49.88	9.77
ZmSPL12	AC233751.1_FGP002	1767	5	588	62.87	8.64
ZmSPL13	GRMZM2G460544_P01	1227	2	408	41.90	8.73
ZmSPL14	GRMZM2G098557_P01	3339	9	1112	121.81	7.54
ZmSPL15	GRMZM2G168229_P01	1221	2	406	42.60	8.88
ZmSPL16	GRMZM2G163813_P02	996	3	331	36.18	8.98
ZmSPL17	GRMZM2G065451_P01	1449	3	482	50.72	9.01
ZmSPL18	GRMZM2G138421_P01	2886	10	961	105.08	5.78
ZmSPL19	GRMZM2G414805_P03	1320	3	439	47.11	9.10
ZmSPL20	GRMZM2G097275_P01	1440	3	479	50.86	9.15
ZmSPL21	GRMZM2G126827_P01	999	3	332	36.51	8.96
ZmSPL22	GRMZM2G111136_P01	1323	2	440	46.00	6.70
ZmSPL23	GRMZM2G156621_P01	999	3	332	36.51	8.96
ZmSPL24	GRMZM2G101499_P01	1305	2	434	46.04	9.14
ZmSPL25	GRMZM2G307588_P01	1137	2	378	39.60	9.33
ZmSPL26	GRMZM2G067624_P02	621	1	206	21.04	9.75
ZmSPL27	GRMZM2G081127_P01	2586	11	861	96.56	7.48
ZmSPL28	GRMZM2G109354_P01	2553	9	850	94.02	5.41
ZmSPL29	GRMZM2G371033_P01	930	2	309	33.66	8.84
ZmSPL30	GRMZM2G148467_P01	1353	2	450	47.01	9.31
ZmSPL31	GRMZM2G058588_P01	1191	2	396	43.27	7.81

[a] Designated name of maize *SPL* genes in this study.

[b]Length of open reading frame in base pairs.

[c]Length (number of amino acids), molecular weight (kilodaltons), and isoelectric point (pI) of the deduced polypeptides.
