# Supplementary material for: Comparative genome analysis of the SPL gene family reveals novel evolutionary features in maize
Source: Genet Mol Biol. 2019 Jul 1;42(2):380–94. doi: 10.1590/1678-4685-GMB-2017-0144 (PMC6726161; doi:10.1590/1678-4685-GMB-2017-0144)
Supplement: Supplementary file 5 [file 1415-4757-GMB-1678-4685-GMB-2017-0144-suppl1.pdf]

Supplementary Material to "Comparative genome analysis of the SPL gene family reveals novel evolutionary features in maize"

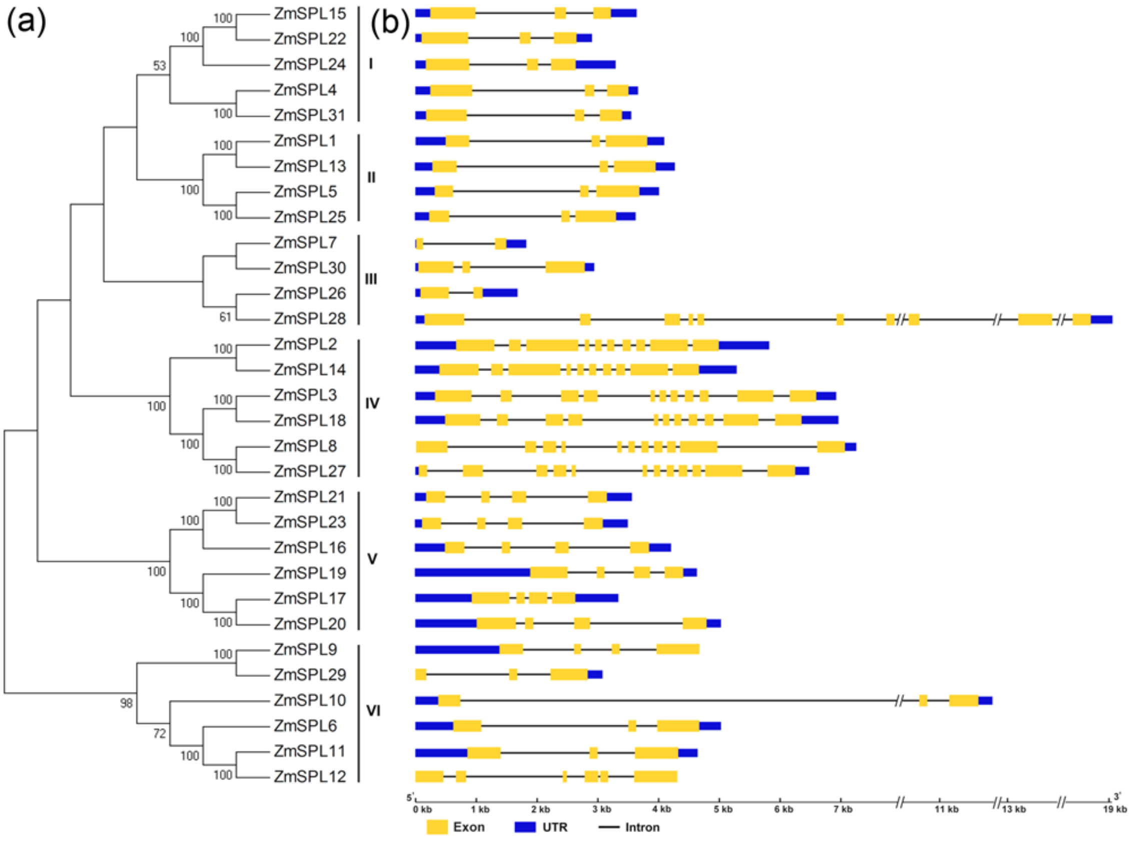

Figure S1 - Phylogenetic relationships and gene structure of the *ZmSPL*s. (a) The unrooted tree was generated using MEGA4.0 program with the NJ method. Bootstrap values above 50% from 1,000 replicates are shown at each node. (b) Yellow rectangles and thin lines represent exons and introns, respectively. Blue lines represent the untranslated regions (UTRs).
