# Supplementary material for: Comparative genome analysis of the SPL gene family reveals novel evolutionary features in maize
Source: Genet Mol Biol. 2019 Jul 1;42(2):380–94. doi: 10.1590/1678-4685-GMB-2017-0144 (PMC6726161; doi:10.1590/1678-4685-GMB-2017-0144)
Supplement: Supplementary file 6 [file 1415-4757-GMB-1678-4685-GMB-2017-0144-suppl8.pdf]

Genetics and Molecular Biology

Supplementary Material to "Comparative genome analysis of the SPL gene family reveals novel evolutionary features in maize"

Table S5 - Detailed information of the 20 motifs identified in *ZmSPLs*

Motif	Width	Conserved amino acid sequences
1	41	GQEQRFCQQCSRFHLLAEFDEKKRSCRKRLAGHNRRRRKPQ
2	21	KDYHRRHKVCEMHSKAPVVVV
3	80	TDKIVFKLFGKEPKDFPPNLREQILNWLKHYPTDMESYIRPGCIILTIYLRMPNWMWD ELEENMAQWINKLINSSNDDFW
4	15	PRCQVEGCKADLSRC
5	80	KVYLFTPNMPGPSNITPLHIAATIEDAEDVLDALTDDPQQIGITCWKNARDETGFTPE EYARKRGHNSYIELVQKKIDKM
6	41	YMLSMMAIAAVCVCVGIIMKTLPRVYAMKTFKWEKIEWGPM
7	42	MTKEDVTDYCLIRFPMWRFRWLLVFAMEREWCAVIKMLLDIM
8	112	DVSTKVGTITGQVHLDKSHMFNAIPTLSHGKDEPWPMKGLDMSISASKFDGAADLQ HALSLLSAGACGLPDSVHQTSCIIQFNGASENSSDLHVTHGRNSGPASCADAQHIA
9	57	NYPKILCVTPIAVPYSQTINFIVKGFNLVIPTTKLICTFEGKYIIQEETCMAYENTI
10	200	QTVPSTMDLLGVFPTGFATSTPETNTSQSQGSSDSSGNNKSKSHSTEPVTVVNSHDKS TRDFPAAGFMRSNSTQESRPHIYKQTEQETRPYLSLQLFGSTEEDFPPKMDSINKYLSS ESSNPLDERSPSSSPPITRKFFPIHSVDEEVRHPHITDYGEDATMGEVSTNQAWCAPPL DLFKDLERPIENGSPPNPGYQSCY
11	17	VIGLKLGKRTYFEDACG
12	159	NYLLLSLIGICANMNSDNVQNANGQELLSTLLKNLGSVAKSLEPKELCKFLEAYQSL QNGLNAGTSGIANGTEEAAGPSNSKLPFVNDSHCGQASSSVVPVQSKATIVVTPEPAS CKLKDFDLNDTCNDMEGFEDGQEGSPTPAFKTADSPNCASWMQQ
13	29	RGFIEVEDYGFSNGFFPFIIAEKDVCSEI
14	29	PMEMVLEENLLHTAVKRKSKNMVHFLLRY
15	15	DSNCALSLLSTQPWD
16	20	YRSQHLHFLNELGWLLQRNN
17	33	PDAFTFASARLPSTFYDDRRQISFVWNKAPYSH
18	18	WDPNLWDWDNHAFTATPS
19	78	NQENAANRTQDIVNLITVIARLQGSNVGKVPSIPPIPDKQNLVEIISKINSFNNMTSPDK SPPPEVVDLDTLQEQREQ
20	24	QEPGRFRSFMLDFSYPRVPSAMRD
