# Supplementary material for: Comparative genome analysis of the SPL gene family reveals novel evolutionary features in maize
Source: Genet Mol Biol. 2019 Jul 1;42(2):380–94. doi: 10.1590/1678-4685-GMB-2017-0144 (PMC6726161; doi:10.1590/1678-4685-GMB-2017-0144)
Supplement: Supplementary file 7 [file 1415-4757-GMB-1678-4685-GMB-2017-0144-suppl2.pdf]

Supplementary Material to "Comparative genome analysis of the SPL gene family reveals novel evolutionary features in maize"

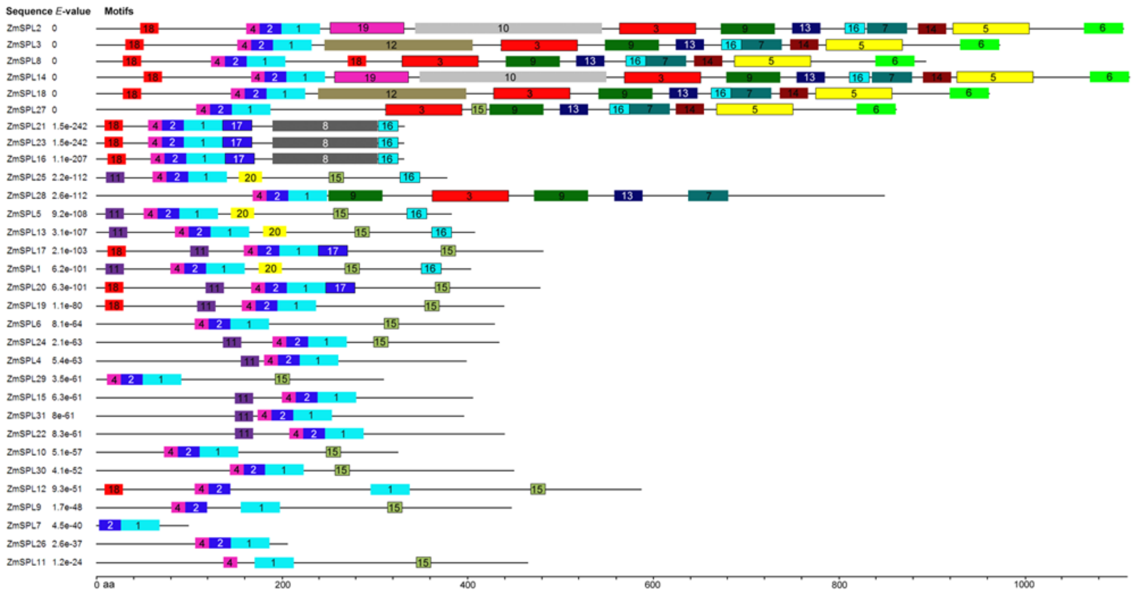

Figure S2 - Distribution of conserved motifs identified in the putative SPL proteins. Twenty motifs were identified by the online MEME server. Different motifs are highlighted with different colors. The length of each motif can be evaluated by the scale at the bottom.
