# Supplementary material for: Comparative genome analysis of the SPL gene family reveals novel evolutionary features in maize
Source: Genet Mol Biol. 2019 Jul 1;42(2):380–94. doi: 10.1590/1678-4685-GMB-2017-0144 (PMC6726161; doi:10.1590/1678-4685-GMB-2017-0144)
Supplement: Supplementary file 8 [file 1415-4757-GMB-1678-4685-GMB-2017-0144-suppl3.pdf]

Supplementary Material to "Comparative genome analysis of the SPL gene family reveals novel evolutionary features in maize"

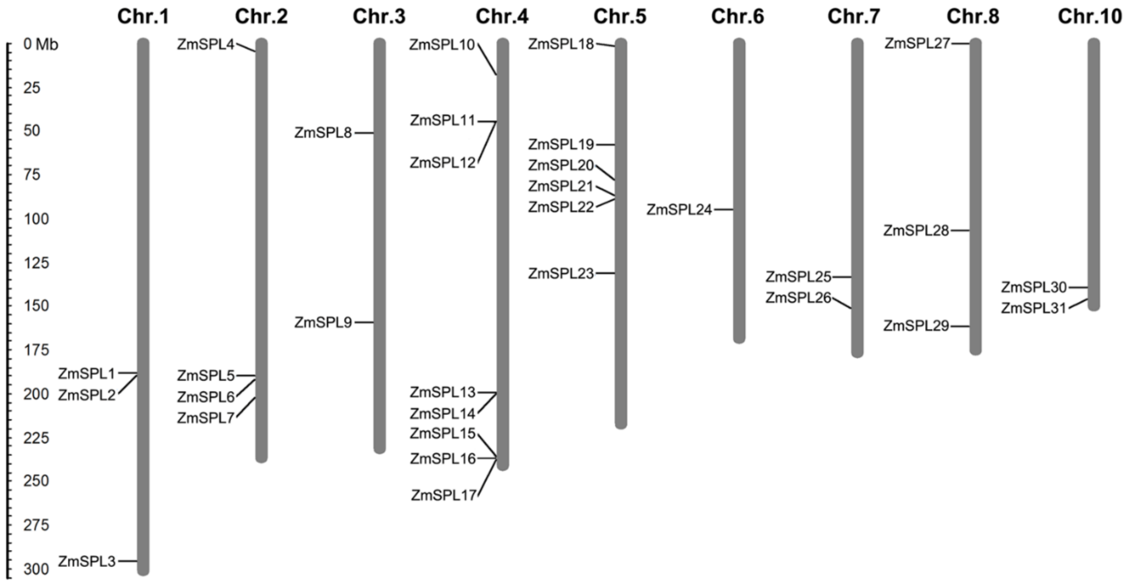

Figure S3 - Chromosomal locations of *ZmSPLs* on the 10 maize chromosomes. The scale on the left is in megabases (Mb). Chromosome numbers are shown on the top of each bar. The names on the left side of each chromosome correspond to the location of each ZmSPL.
